# Use of the traditional Chinese medicine “compound healthy ear agent” to protect against age-related hearing loss in mice: A proteomics study

**DOI:** 10.1016/j.heliyon.2024.e26914

**Published:** 2024-02-25

**Authors:** Weijun Xuan, Liyi Huang, Yi Xuan, Sizhong Chen, Junbo Tang, Yulong Wei, Xu Pan, Michael R. Hamblin

**Affiliations:** a Department of Otorhinolaryngology, Head and Neck Surgery, First Clinical Medical College and Hospital, Guangxi University of Chinese Medicine, Nanning, China; b Department of Otorhinolaryngology, Head and Neck Surgery, International Zhuang Medical Hospital of Guangxi, Guangxi University of Chinese Medicine, Nanning, China; c Department of Infection, The First Affiliated Hospital, Guangxi Medical University, Nanning, China; d School of Engineering, Tufts University, Medford, MA, 02155, USA; e Department of Otorhinolaryngology, Renai Branch Hospital, Guangxi University of Chinese Medicine, Nanning, China; f Department of Pharmaceutical Manufacturing, Ruikang Clinical Medical College, Guangxi University of Chinese Medicine, Nanning, China; g Laser Research Centre, Faculty of Health Science, University of Johannesburg, Doornfontein 2028, South Africa

**Keywords:** Proteomics, C57BL/6J mice, Presbycusis, Traditional Chinese medicine, Cochlear hair cell degeneration

## Abstract

**Background:**

Previous studies have shown that the traditional Chinese medicine (TCM) called “compound healthy ear agent” (CHEA) had anti-apoptosis effects in cochlear hair cells and spiral ganglion neurons, and could protect mice hearing against presbycusis or age-related hearing loss (AHL), as well as aminoglycoside antibiotic-induced ototoxicity. Because its mechanisms of action are still unclear, we investigated the mechanism of action of CHEA against AHL in mice using proteomics techniques.

**Methods:**

Eighteen C57BL/6J mice at 1 month of age were randomly divided into three groups: (A) drinking water until 2 months of age, K2M); (B) drinking water until 7 months of age to induce AHL, K7M; (C) drinking water containing CHEA daily until 7 months of age as treatment group, Z7M. At 2 or 7 months mice were sacrificed and their cochleae were removed for proteomics analysis.

**Results:**

The numbers of proteins with a false discovery rate (FDR) < 1% were respectively 5873 for qualitative and 5492 for quantitative statistics. The numbers of proteins with differential enrichment at least 1.5-fold (p < 0.05) were respectively 351 for K7M vs K2M groups, 52 for Z7M vs K7M groups, 264 for Z7M vs K2M groups. The differentially expressed proteins in the Z7M group were involved in synaptic molecular transmission, energy metabolism, immune response, antioxidant defenses, and anti-apoptosis.

**Conclusion:**

The TCM CHEA played a protective role against AHL in mice by regulating the expression of specific proteins and genes in cochlear hair cells and spiral ganglion neurons. Besides the pathways expected to be involved (antioxidant and anti-apoptosis), proteins related to immune response is a new finding of the present study.

## 1 Introduction

Age-related hearing loss (AHL) or presbycusis is a type of deafness that presents as a bilateral, symmetrical hearing loss (HL) which begins with the inability to hear high frequencies [1]. Pathologically it involves progressive, bilateral and symmetrical degeneration of the inner ear structures [2]. AHL affects >50% of adults by age 75 years, and nearly all adults over 90 years of age [3]. The etiology and pathology of AHL are quite complex, and many aspects are still unclear. A series of previous studies have shown that the traditional Chinese medicine “compound healthy ear agent” (CHEA) had significant anti-apoptotic effects in cochlear hair cells and spiral ganglion neurons, and could protect the hearing of mice with AHL [[4], [5], [6], [7], [8]]. CHEA also known as “Jian Er”, is an herbal preparation mainly containing *Radix puerariae, Salvia miltiorrhiza, Astragalus membranaceus,* and Drynaria fortune. Further studies found that its mechanisms of action were related to enhancing antioxidant enzymes, reducing the formation of reactive oxygen species (ROS), protecting mitochondrial function, and increasing the expression of nerve growth factors [9,10].

For over a decade, we have repeatedly studied the C57BL/6J mouse model of AHL in China and found that the loss of hair cells had occurred at the hook region and its vicinity in the cochlear basal turn by 3 months of age. By 7 months of age, additional hair cells were missing in the front and middle segments of the cochlear basal turn, and high-frequency hearing loss could be detected. The spiral ganglion cells and nerve fibers were also damaged in the cochlear basal turn by 7 months of age. However, in 2-month-old mice the hair cells were not damaged, and the mouse hearing thresholds remained within the normal range. As shown by observation from 3 months to 24 months of age, the missing hair cells progressively increased in number from the basal turn to the apical turn, The hearing loss also progressed from high frequency to low frequency over this period. Therefore 7-month-old mice were chosen as a relatively short period of time to provide alterations in hair cell morphology and audiology findings, as we have reported in our previous drug interventional research [11]. This choice could also shorten the observation time and save manpower and resources. Therefore, animals of 2, 3 and 7 months of age were considered to be suitable models for studying traditional Chinese medicine in the treatment of AHL [12].

In order to further investigate the etiology and pathology of AHL and understand the mechanism of the pharmacodynamic effects of CHEA in the prevention and treatment of AHL, we employed 7 months old C57BL/6J mice as an animal model of AHL as described in previous studies. After 7 months of treatment with CHEA in drinking water, we removed the cochleae and analyzed them using proteomics technology. The goal was to explore the biological effects of CHEA on the up-regulation or down-regulation within a wide range of specific proteins.

## 2 Materials and methods

### 2.1 Experimental animals

Eighteen SPF 1-month-old newly weaned healthy C57BL/6J mice, half male and half female, weighing 16–18 g, were provided by Beijing Weitong Lihua Laboratory Animal Technology Co., LTD. (SCXK Beijing 2012-000), and kept in a SPF animal laboratory (SYXK Guangxi 2019-000). All operations were in accordance with the ethical requirements for experimental animals of Guangxi University of Traditional Chinese Medicine (Approval No.: IACUC 20161015).

### 2.2 Experimental groups

18C57BL/6J mice were selected from the same batch of 60 animals, and were randomly divided into three groups of six mice each with half female and half male. Six animals in the first group drank tap water from weaning (1 month of age) until 7 months of age as the AHL group (K7M group); six animals in the second group drank tap water containing CHEA daily, from 1 month until 7 months of age as the treatment group (Z7M group). In addition, an additional six untreated C57BL/6J mice drank tap water until 2 months of age (K2M group) and were used as a control group before disease development. Mice were sacrificed at 2 months of age for K2M group and at 7 months of age for K7M and Z7M groups, by decapitation under deep ketamine/xylazine anesthesia. Cochleae were prepared and evaluated as described in detail in previous publications [6,[13], [14], [15], [16]]. The temporal bones were removed and the cochleae were surgically removed (2 cochlear samples per animal). The tissue samples were snap frozen in liquid nitrogen and stored at −80 °C. Three animals (2 male and 1 female) were randomly selected from each group to be used for initial measurement of proteomics. The remaining three animals (1 male and 2 female) per group were used to repeat the proteomics assays as a quality control. The second repetition of the proteomics assay was in close agreement with the first determination, and the data are not reported separately.

### 2.3 Preparation of CHEA

The traditional Chinese medicine CHEA is patented in China (Patent No. ZL2013104661403) for the prevention and treatment of sensorineural deafness. CHEA is composed mainly of the following herbs: *Radix puerariae, Salvia miltiorrhiza, Astragalus membranaceus,* and Drynaria fortune. The chemical components of CHEA are comprised mainly of isoflavones, lipid-soluble diterpenoid quinones, water-soluble phenolic acids, astragalus polysaccharide, saponins, flavonoids, triterpenes, phenolic acids, etc. CHEA was manufactured in the Pharmaceutical Factory of Ruikang Medical College in Guangxi University of Chinese Medicine. These botanical materials were successively treated by cooking in water, filtering, concentrating, drying, pulverizing, and passing through a mesh to produce a powder according to the methods described in the Patent previously used to prepare this herbal medicine in capsules. One gram of the final CHEA powder was equal to 6.63 g of the raw herbal materials. By applying the rule of dosage equivalence between a mouse and an adult human [17], the dosage for mice was calculated to be 1.83 g/kg/day. Powder (0.05 g) was added to 5 mL of water, and then provided to the animals in their water bottles from 1 month of age to 4 months of age. After that, mice were daily administered the calculated dosage (per body weight) by gavage needle, from 4 months of age to 7 months of age. These animals received the calculated dosage in drinking water each day without any other source of water.

### 2.4 Reagents and instruments

The main reagents employed in the experiments are listed in Table 1.

**Table 1:** Main reagents.

Reagent Name	Producer	Product number	Batch number
TMT10 labeling kit	ThermoScientific	90110	N-TD264171
Hydroxylamine	ThermoFisher	90115	PD199190
BCA Kit	ThermoScientific	23227	UB276927
Mass spectrometry grade acetonitrile	ThermoScientific	A955-4	168772
Mass spectrometry water	ThermoScientific	W6-4	180416
Lysis solution	Biyuntian Biotechnology Research Institute	p0013g	092918190103
PMSF	SangonBiotech	A610425-0005	E809BA0022
Na2HPO4▪12H2O	SangonBiotech	A501725-0500	E719BA0012
Na2H2PO4▪H2O	SangonBiotech	A100823-0100	E329BA0012
NaCl	SangonBiotech	A501218-0001	E502BA0012
40% Acr-Bis	SangonBiotech	B546014-0500	EB02KA0062
Tris-HCl/pH6.8/pH8.8	SangonBiotech	B546020-0250/B546019-0250	E227KA7816/EA18KA9854
APS	SangonBiotech	A600072-0025	DC20BA1005
TEMED	SangonBiotech	A100761-0025	C106BA0030
Tris	SangonBiotech	A600194-0500	F117BA0002
DTT	SangonBiotech	A620058-0005	C729BA0004
Glycerin	SangonBiotech	A100854-0500	BA19BA0001
Bromophenol blue	SangonBiotech	A100449-0005	EC05BA0011
TFA	SangonBiotech	TS4295-013	13020696
Urea	SangonBiotech	A600148-0002	BB06BA0025
IAA	SangonBiotech	A600539-0005	EA16BA0005
G-250	SangonBiotech	A600077-0025	E809BA0014
Glycine	Sinopharm Chemical Reagent	17-1323-01	171204107
SDS	Sinopharm Chemical Reagent	17-1313-01	15J070011
Phosphate buffer	Sinopharm Chemical Reagent	10015418	LOT: 20150706
TEAB	SIGMA	T7408-500 mL	BCBX6381
Absolute ethyl alcohol	GENERAL-REAGENT	G73537B	P1346753
Isopropyl alcohol	ThermoScientific	A451-4	182060
Trypsin	Beijing hualishi Technology	HLS try001c	no20190820c
Formic acid	CNW Technologies GmbH	CAS:64-18-6	172433

The main instruments employed in the experiments are listed in Table 2.

**Table 2:** Main instruments.

Instrument Name	Manufacturer	Model number
Q Exactive Mass spectrometer	ThermoFisher	IQLAAEGAAPFALGMBDK
Easy-nLC 1200 Liquid phase system	ThermoFisher	Easy-nLC 1200
High performance liquid chromatograph	Agilent	Agilent 1100 series
Orbitrap Fusion	ThermoFisher	IQLAAEGAAPFADBMBCX
Q Exactive HF	ThermoFisher	IQLAAEGAAPFALGMBFZ
Desktop refrigerated centrifuge	Shanghai Luxianyi centrifuge instrument	TGL-16A
Ultrasonic cell disruptor	Ningbo Xinzhi Biotechnology	JY98-IIIN
SDS-PAGE Gel electrophoresis apparatus	Beijing six One Instrument Factory	DYY-6C
Enzyme-labeled instrument	Shanghai Kehua Experimental System	ST-360
ImageScanner	EPSON	ES-1000G
Electronic balance	Shanghai Yueping Scientific Instrument	FA2004B
Freeze drying apparatus	Ningbo Xinzhi Biotechnology	SCIENTZ-10 N

### 2.5 Proteomics methods

The TMTTM (Tandem Mass Tags) in vitro labeling technology developed by ThermoFisher was used for the proteomics study. The brief steps were as follows.

(1) Determination of protein concentration. The frozen sample was added to 300 μL of sample lysis buffer containing the protease inhibitor PMSF, at a final concentration of 1 mM. The samples were subjected to ultrasonic tissue disruption on ice at 80 W power, 1 s ultrasound bursts were applied 90 times in 3 min. The supernatant was obtained by centrifugation at 12000 rpm for 10 min at room temperature and centrifuged again. The supernatant contained the total protein contents of the sample, which was measured for protein concentration and then stored at −80 °C for later use. The protein concentration was determined by the BCA method (bicinchoninic acid). Under alkaline conditions when BCA binds to proteins, Cu2+ is reduced to a Cu+ chelate with two BCA molecules, and the working reagent forms a purple complex from the original apple green. The water-soluble complex shown a good linear relationship of absorbance at 562 nm and protein concentration over a wide range. According to the instructions of the BCA reagent kit, the required volume of buffer solution was prepared with buffers A: B (50:1 v/v). The protein solution was diluted with ultrapure water to within the working range of the standard curve. A clean 96 well plate was aliquoted with a gradient of BSA: 0, 1, 2, 4, 8, 12, 16, 20 μL of a 1 mg/mL solution. The corresponding volume of ultrapure water was added until the total volume was 20 μL. Samples of the protein test solution were adjusted to 20 μL and added to the wells in triplicate. Then 200 μL chromogenic solution was added to each well and reacted at 37 °C for 30 min. The absorbance values were measured at 562 nm using a microplate reader. The protein concentration was obtained using the standard curve.
(2) SDS-polyacrylamide gel electrophoresis (12% SDS-PAGE) was carried out with 10 μg protein taken from each sample. The gel was stained by Coomassie brilliant blue, and quantified using an ImageScanner in full-color scanning mode and an optical density value of 300 dpi.
(3) Trypsin enzymatic hydrolysis and labeling. After proteins were quantified, 100 μg of each sample in an ultrafiltration tube plus 120 μL reducing agent buffer (10 mM DTT, 8 M urea, 100 mM TEAB, pH 8.0) was incubated at 60 °C for 1 h. Trypsin hydrolysis and TMT labeling was performed according to the manufacturer's protocol. 8 μL of 5% hydroxylamine was used to terminate the reaction for 15 min, and then the samples were lyophilized and stored at −80 °C.
(4)Reverse phase chromatographic separation was performed using an Agilent 1100 HPLC chromatographic column: Agilent Zorbax Extend C18 narrow diameter column, 2.1 × 150 mm, 5 μm. Detection wavelength: UV 210 nm and 280 nm. Mobile phase A: ACN-H2O (2:98, v/v). Mobile phase B: ACN-H_2_O(90:10, v/v). Flow rate: 300 μL/min. Gradient elution conditions: 0–8 min, 98% A; 8∼8.01 min, 98%–95% A; 8.01–48 min, 95%–75% A; 48–60 min, 75∼60% A; 60∼60.01 min, 60∼10% A; 60.01–70 min, 10% A; 70∼70.01 min, 10–98% A; 70.01–75 min, 98% A. Samples were collected each minute over 8–60 min.
(5)After collection, the samples were lyophilized and stored for LC-MS. Chromatographic conditions: flow rate of 300 nL/min to Acclaim PepMap 100 100 μm × 2 cm column (RP-C18, Thermo Fisher), and then followed by an analytical column Acclaim PepMap RSLC, 75 μm × 15 cm (RP-C18, Thermo Fisher). Mobile phase A: H_2_O-FA (99.9:0.1, v/v); Mobile phase B: ACN–H_2_O–FA (80:19.9:0.1, v/v/v); Gradient elution conditions: 0–40 min, 5–30% B; 40–54 min, 30–50% B; 54–55 min, 50–100% B; 55–60 min, 100%B. Mass spectrometry conditions: resolution ratio of Class A MS was set at 70000, and the automatic gain control value was set as 1e6. The mass spectrum was scanned over a *m*/*z* range of 300–1600, and MS/MS scanning was performed on 10 of the highest peaks. Collection of all MS/MS spectra used energetic collision and splitting using data-dependent positive ion mode. The collision energy was set at 32 eV, the resolution ratio of MS/MS was set at 35000, the automatic gain control was set at 2e5, the maximum ion accumulation time was 80 ms, the dynamic exclusion time was set at 30 s.

### 2.6 Data analysis

The Proteome Discoverer™ 2.2 (Thermo Company, USA) software was used for analysis. We employed the mouse database of UniProt. The false discovery rate (FDR) for peptide identification was calculated by dividing the number of expected false positive identifications by the total number of target identifications and was always less than 1%. The retrieved results were screened for credible proteins according to the criteria of Score Sequest HT > 0, unique peptide ≥1, and the blank values were removed. Based on the screened credible proteins (multiple repetitions were analyzed on the basis of proteins that could be identified), the fold change (FC) values were calculated and p-values compared by a two-tailed Students t-test. With a FC > 1.5 or a FC < 0.66 and a p-value <0.05 as the criteria, significant proteins were identified. The cloud platform of OmicsBean (http://www.omicsbean.cn/) was used for omics data integration and analysis, and Gene Ontology (GO, http://geneontology.org/) for functional annotation, enrichment and KEGG pathway analysis (https://www.genome.jp/kegg/pathway.html) on the differentially expressed proteins. The obtained data were uploaded to iProx (https://www.iprox.cn/).

## 3 Results

**Fig. 1:**
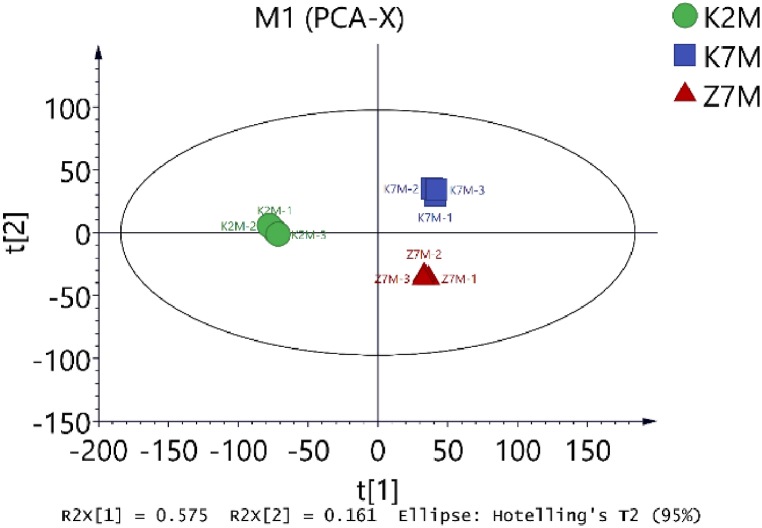
PCA analysis for quality control of data. The protein expression in three groups was analyzed by principal component analysis (PCA). Each point in the figure represents a repetition within each group. Different colors and shapes represent different groups. 95% confidence ellipse using Hotelling T-squared. M1 (PCA-X) is a standard parameter generated by Proteome DiscovererTM 2.2 software. (For interpretation of the references to color in this figure legend, the reader is referred to the Web version of this article.)

**Fig. 2:**
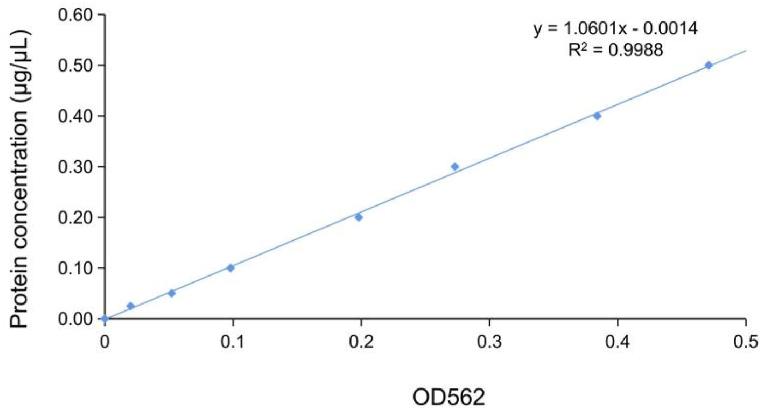
Standard curve for BCA protein assay.

**Fig. 3:**
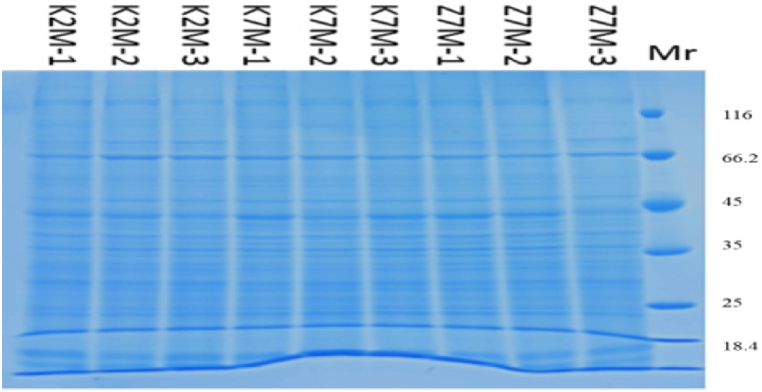
Example of SDS-PAGE protein separation.

We used PCA analysis for quality control of the data. Each point in Fig. 1 represents a repetition of a group experiment, with different colors and shapes distinguishing different groups. The protein quantification standard curve is shown in Fig. 2. The molecular weights of the proteins were determined by SDS-PAGE [Fig. 3]. The relative amounts of the peptides were obtained by calculating the peak area ratios from HPLC chromatograms. After LC-MS/MS detection and the database search, the number of qualitatively different proteins was 5873. Among these, the number of proteins with FDR<1% and Abundance>1 detected was 5492. The qualitative and quantitative analysis was performed with the Shotgun proteomics method, which first used a protease to cleave the protein into peptide segments, and then used chromatography tandem mass spectrometry to detect the peptides. Through matching the peptide segment map with the theoretical map in the database, the proteins were qualitatively identified, and then the proteins were relatively quantified by the peak intensity in mass spectrometry.

### 3.1 Volcano plots and heat maps

**Fig. 4:**
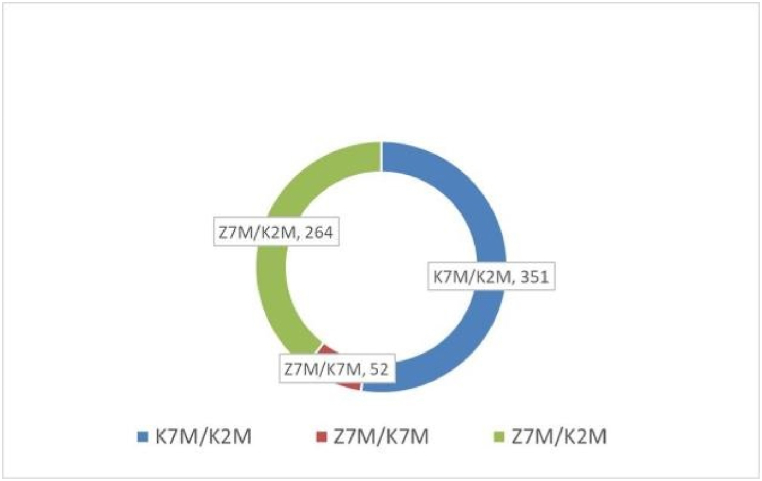
Numbers of differentially expressed proteins between the three comparison groups. The numbers of differentially expressed proteins (1.5 fold increased or 0.66 fold decreased, p < 0.05 using an unpaired *t*-test) are shown in each comparison group.

**Fig. 5:**
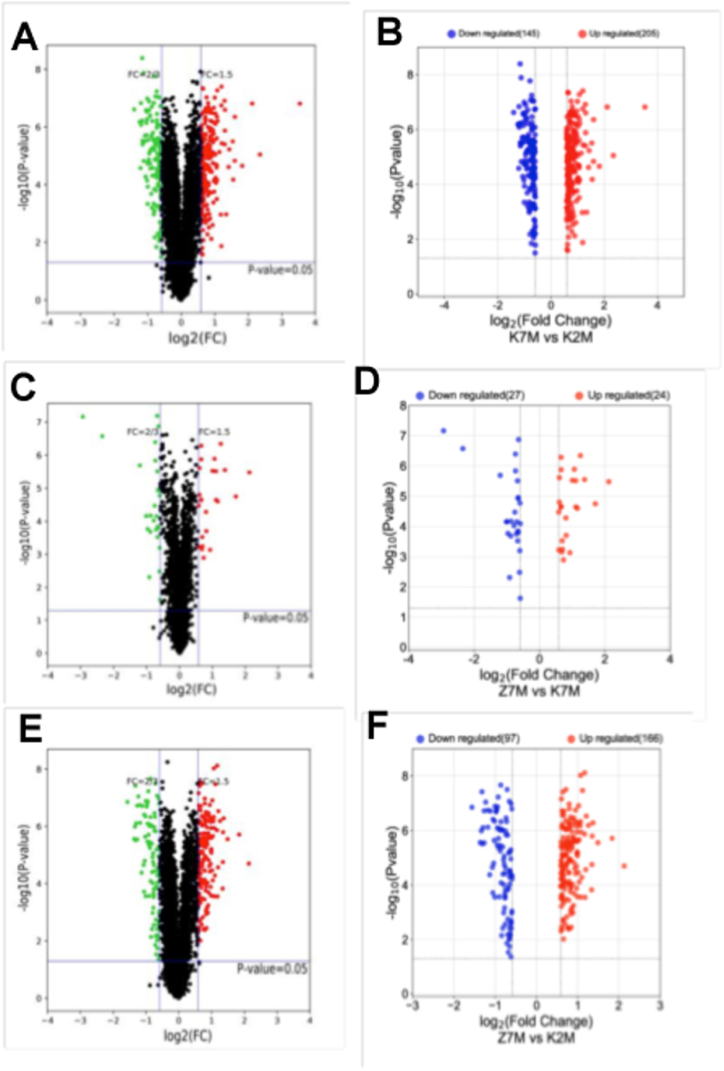
Proteomics volcano plots for the three comparison groups. The abscissa of the volcano plot is log 2 (FC). The farther the value was from the zero point, the greater the difference. The right side of each plot shows up-regulated proteins, and the left side shows down-regulated proteins. The ordinate is - log 10 (P-value). The green or blue dots show significantly down-regulated proteins, while the red dots show significantly up-regulated proteins, and the black dots show non-significantly differentially expressed proteins. Plots on the left (A, C, E) show all proteins while plots on the right (B, D, F) show only significantly different proteins. (A, B) are K7M vs K2M; (C, D) are Z7M vs K7M; (E, F) are Z7M vs K2M. (For interpretation of the references to color in this figure legend, the reader is referred to the Web version of this article.)

**Fig. 6:**
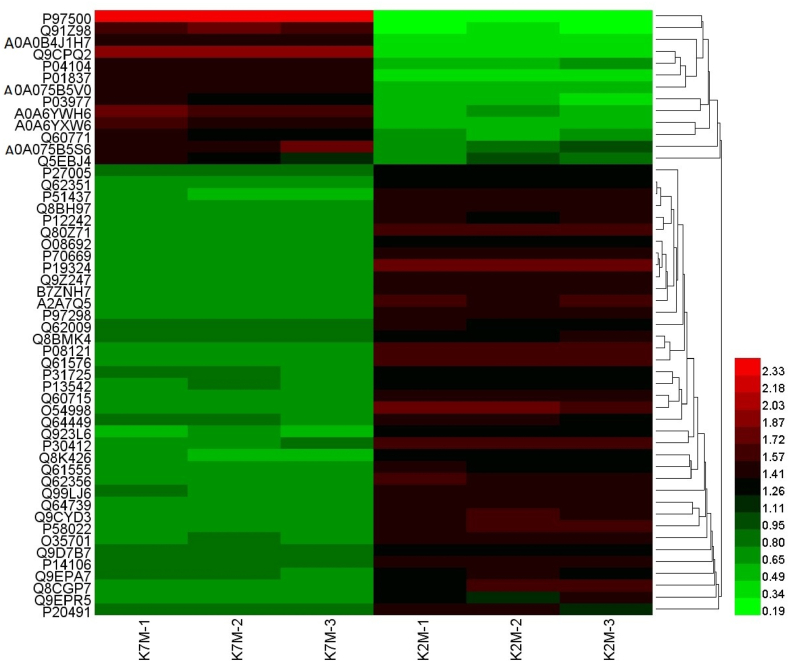
Proteomics heat map for the K7M vs K2M comparison group. K7M-1, etc refer to individual mice from each group of three mice.

**Fig. 7:**
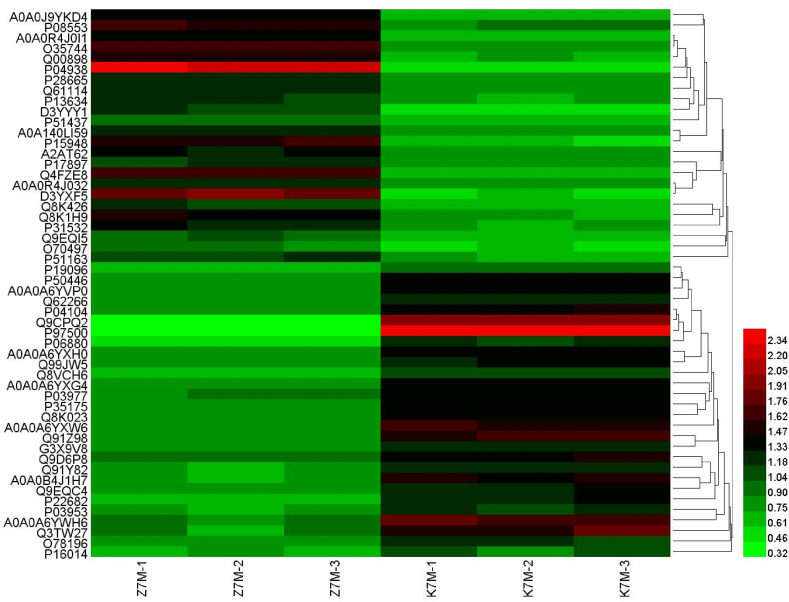
Proteomics heat map for the Z7M vs K7M comparison group. K7M-1, etc refer to individual mice from each group of three mice.

**Fig. 8:**
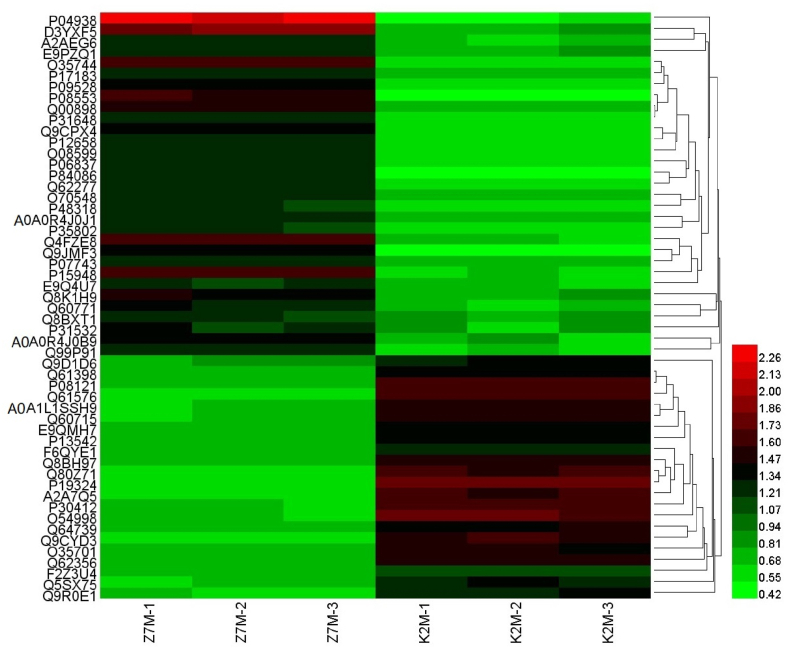
Proteomics heat map for the Z7M vs K2M comparison group. Z7M-1, etc refer to individual mice from each group of three mice.

The proteins were identified and differentially expressed proteins were screened from the original data using the following criteria as recommended by Proteome DiscovererTM 2.2 software. The screening criteria were: (1) Score Sequence HT was greater than 0; (2) the number of peptide segments was greater or equal to 1; (3) data with blank expression values were deleted. The numbers of the differentially expressed proteins in each group are shown in Fig. 4. Next the functional analysis of biological information was carried out based on the differentially expressed proteins. The volcano plots of proteomics of the three different comparison groups (K2M vs K7M; K7M vs Z7M; K2M vs Z7M) are shown in Fig. 5A–F. The clustering heat maps of the three comparison groups contain specifically named differentially expressed proteins (Fig. 6, Fig. 7, Fig. 8). The numbers of differentially expressed proteins in the K7M/K2M or Z7M/K2M comparison groups were higher than the number in the Z7M/K7M comparison group.

### 3.2 Gene ontology

**Fig. 9:**
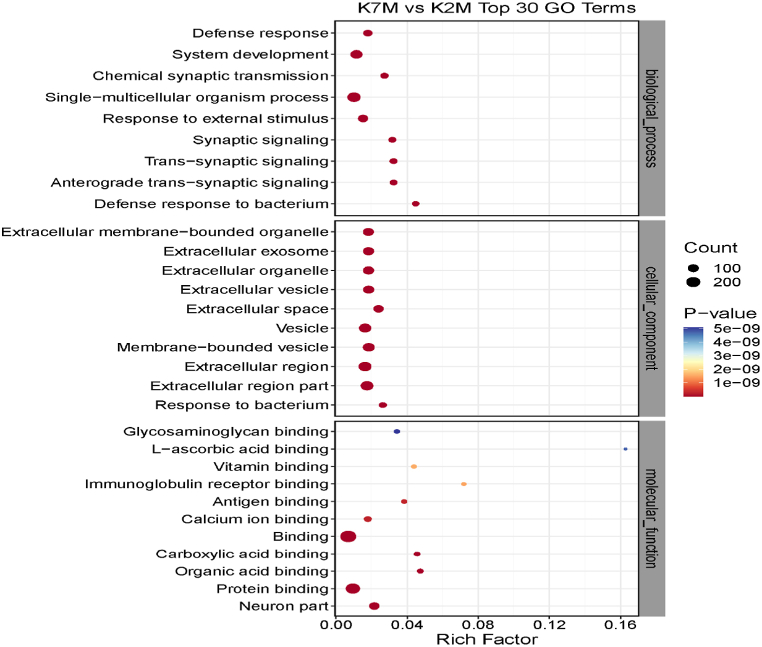
Proteomics GO enrichment analysis for the K7M vs K2M comparison group (Top 30 GO Terms). The bubble color represents the respective P values, which gradually changes from red to blue indicating the P value is getting smaller. The bubble size indicates the number of proteins involved. Therefore low P values, and larger bubbles with dark red color indicate the most important findings. (For interpretation of the references to color in this figure legend, the reader is referred to the Web version of this article.)

**Fig. 10:**
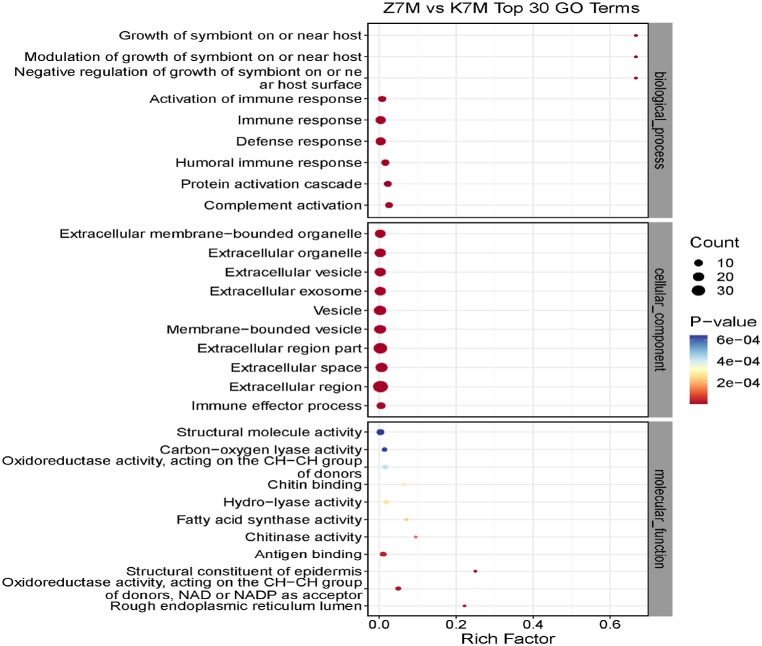
Proteomics GO enrichment analysis for the Z7M vs K7M comparison group (Top 30 GO Terms). See legend to Fig. 9.

**Fig. 11:**
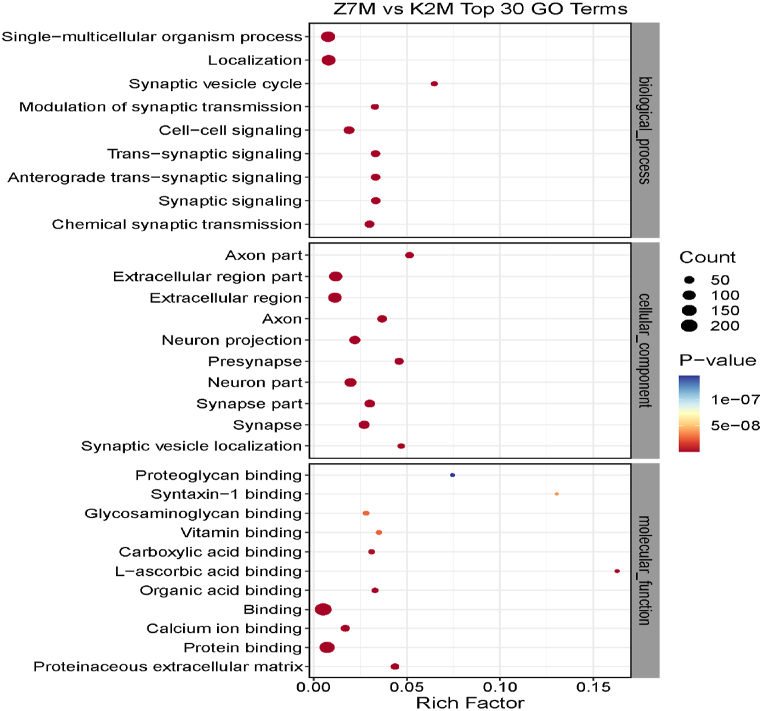
Proteomics GO enrichment analysis for the Z7M vs K2M comparison group (Top 30 GO Terms). See legend to Fig. 9.

The GO results of the three comparison groups showed that the significant differentially expressed proteins were distributed across ten fields of molecular biology, here classified into biological processes, cellular components, and molecular functions (Fig. 9, Fig. 10, Fig. 11) and Table 3, Table 4, Table 5.

**Table 3:** Shows the differentially expressed proteins in the K7M/K2M comparison group.

Biological processes	Cellular components	Molecular functions
Defense response	Extracellular membrane-bounded organelle	Glycosaminoglycan binding
System development	Extracellular exosome	l-ascorbic acid binding
Chemical synaptic transmission	Extracellular organelle	Vitamin binding
Single-multicellular organism process	Extracellular vesicle	Immunoglobulin receptor binding
Response to external stimulus	Extracellular space;	Antigen binding
Synaptic signaling	Membrane-bound vesicle	Calcium ion binding
*Trans*-synaptic signaling	Extracellular region	Carboxylic acid binding
Anterograde *trans*-synaptic signaling	Response to bacteria	Organic acid binding
Defense response to bacteria		Protein binding
		Neuronal components

The most pronounced proteins in the K7M/K2M comparison group were related to system development, single-multicellular organisms, organelles, exosomes, vesicles and protein binding.

**Table 4:** Shows the differentially expressed proteins in the Z7M/K7M comparison group.

Biological processes	Cellular components	Molecular functions
Growth of symbiont on or near host	Complement activation	Carbon-oxygen lyase activity
Modulation of growth of symbiont on or near host	Extracellular membrane-bound organelle	Oxidoreductase activity
Negative regulation of growth of symbiont on or near host surface	Extracellular organelle	Acting on the CH–CH group of substrates
Activation of immune response	Extracellular vesicle	Chitin binding
Immune response	Extracellular exosome	Hydro-lyase activity
Defense response	Vesicle	Fatty acid synthesis activity
Humoral immune response	Membrane-bound vesicle	Chitinase activity
Protein activation cascade	Extracellular region	Antigen binding
	Extracellular space;	Structural constituents of epidermis
	Immune effector process	NAD or NADP as acceptor
		Rough endoplasmic reticulum lumen

The most pronounced proteins in the Z7M/K7M comparison group were related to immune response, defense response, protein activation cascade, organelle, vesicle, extracellular exosome, extracellular region, immune effector, various enzyme activities and antigen binding.

**Table 5:** Shows the differentially expressed proteins in the Z7M/K2M comparison group.

Biological processes	Cellular components	Molecular functions
Single-multicellular organism process	Axon	Proteoglycan binding
Synaptic vesicle cycle;	Extracellular region	Syntaxin-1 binding
Modulation of synaptic transmission	Extracellular region	Glycosaminoglycan binding
Cell-cell signaling	Neuron projection	Vitamin binding
*Trans*-synaptic signaling	Presynapse	Carboxylic acid binding
Anterograde *trans*-synaptic signaling	Neuron part	l-ascorbic acid binding
Synaptic signaling	Synapse part;	Organic acid binding
Chemical synaptic transmission	Synapse	Calcium ion binding
	Synaptic vesicle localization	Protein binding
		Extracellular matrix

The most pronounced proteins in the Z7M/K3M comparison group were related to single-multicellular organism process, cell-cell signaling, synaptic signaling, chemical synaptic transmission, axon, extracellular region, neuron projection, synapse, neuron part and protein binding.

### 3.3 KEGG pathway enrichment analysis

**Fig. 12:**
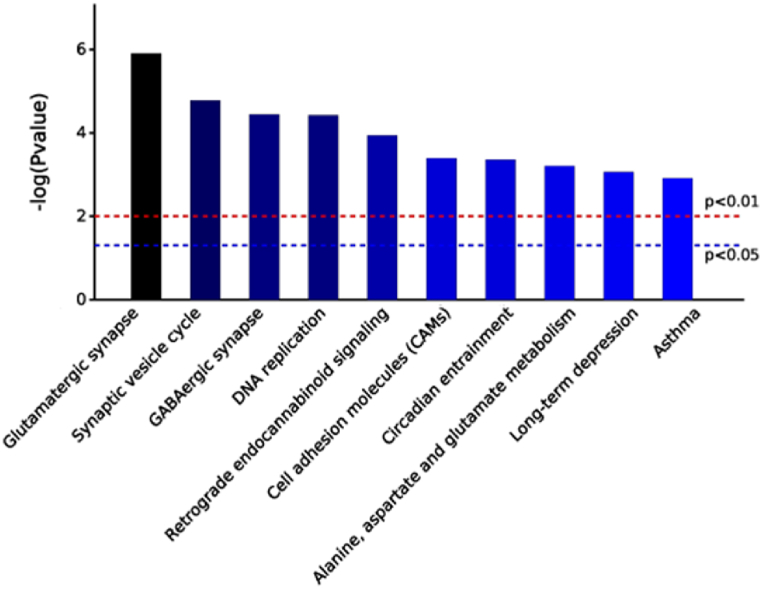
KEGG pathway enrichment analysis for the K7M vs K2M comparison group. Bars show the p values for each significantly altered pathway.

**Fig. 13:**
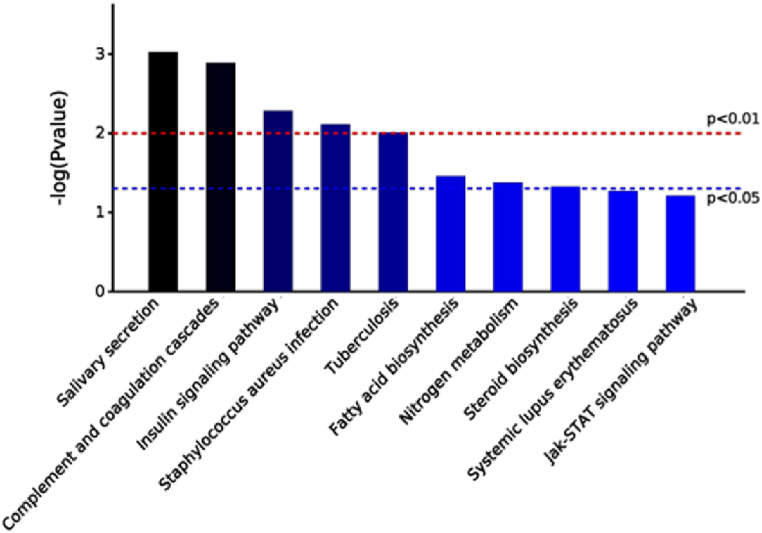
KEGG pathway enrichment analysis for the Z7M vs K7M comparison group. Bars show the p values for each significantly altered pathway.

**Fig. 14:**
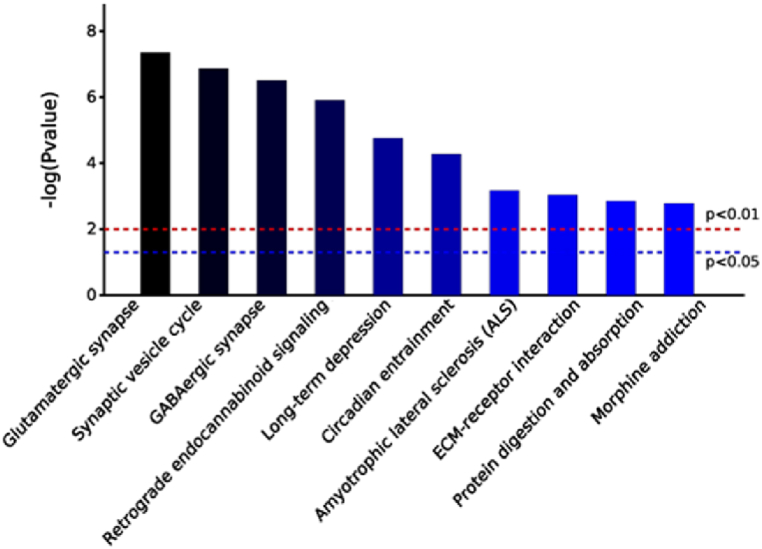
KEGG pathway enrichment analysis for the Z7M vs K2M comparison group. Bars show the p values for each significantly altered pathway.

The top ten enriched pathways with significant differences between the three different comparison groups in KEGG analysis are shown in Fig. 12, Fig. 13, Fig. 14. In the K7M vs K2M comparison group the pathways involved: Glutamatergic synapses; Synaptic vesicle cycles; GABAergic synapses; DNA replication; Retrograde endocannabinoid signaling; Cell adhesion molecules; Circadian entrainment; Alanine, Aspartate and Glutamate metabolism; Long-term depression; Asthma.

In the Z7M vs K7M comparison group, the pathways involved; Salivary secretion; Complement and coagulation cascades; Insulin signaling pathway; *Staphylococcus aureus* infection; Tuberculosis; Fatty acid biosynthesis; Nitrogen metabolism; Steroid biosynthesis; Systemic lupus erythematosus; Jak-STAT signaling pathway.

In the Z7M vs K2M comparison group, the pathways involved: Glutamatergic synapse; Synaptic vesicle cycles; GABAergic synapse; Retrograde endocannabinoid signaling; Long-term depression; Circadian entrainment; Amyotrophic lateral sclerosis; ECM-receptor interaction; Protein digestion and absorption; Morphine addiction.

### 3.4 Protein-protein interactions (PPI)

**Fig. 15:**
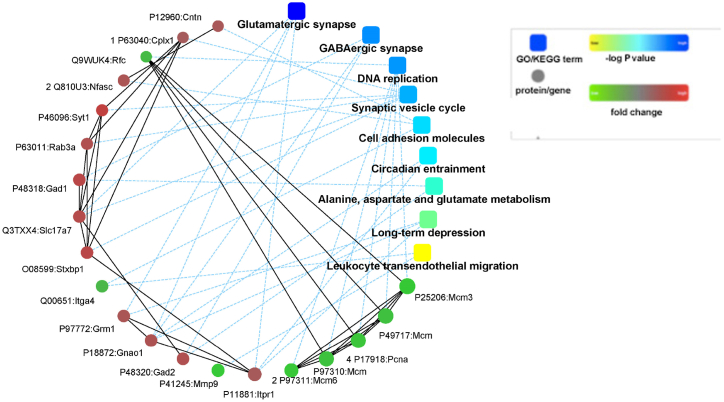
Protein-protein interactions for the K7M vs K2M comparison group (ppiquery/ppigene). The solid lines represent a direct relationship between proteins or genes and pathway groups. The color of the pathway group (yellow to blue) depends on the strength of the association (p value). The color of the gene/protein (green to red) depends on the fold change. The thicker the solid line, the stronger the relationship, and vice versa. The dashed lines represent an indirect relationship between proteins and/or pathway groups. The thinner the dashed line, the weaker the indirect relationship, and vice versa. (For interpretation of the references to color in this figure legend, the reader is referred to the Web version of this article.)

**Fig. 16:**
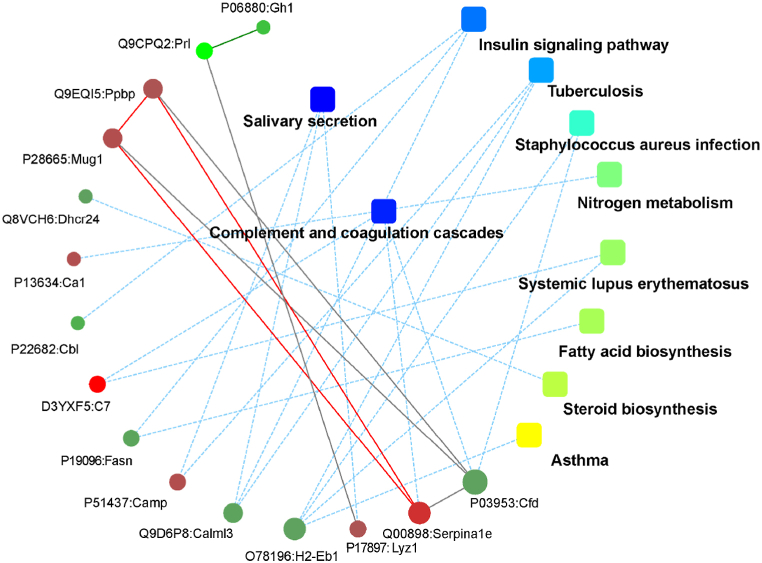
Protein-protein interactions for the Z7M vs K7M comparison group (ppiquery/ppigene). See legend to Fig. 15.

**Fig. 17:**
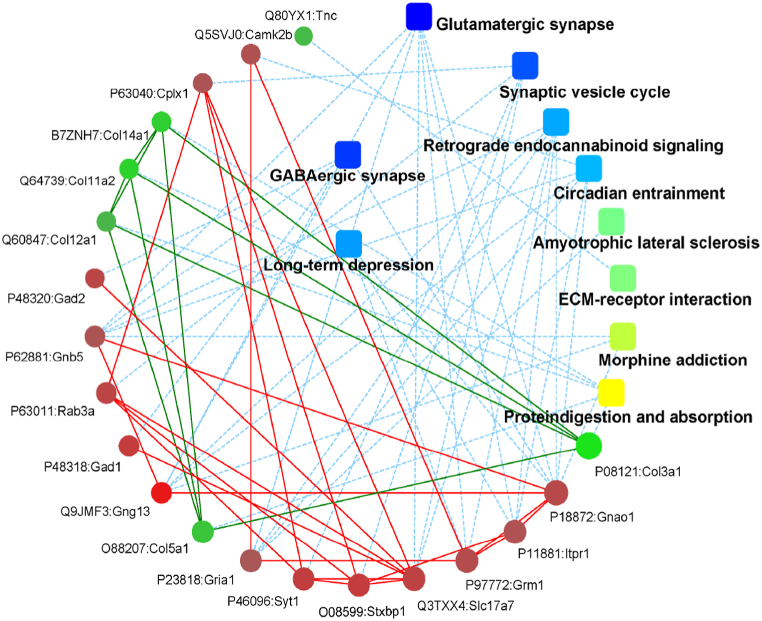
Protein-protein interactions for the Z7M vs K2M comparison group (ppiquery/ppigene). See legend to Fig. 15.

Due to the large number of up-regulated or down-regulated differentially expressed proteins, the determination of relational pathways or network connections between the individual proteins is quite complex to illustrate comparison between groups. A single graph cannot clearly display all of their relationships, so the results of PPI network construction of only a few of the proteins have been selected as examples in Fig. 15, Fig. 16, Fig. 17. In the K7M vs K2M comparison group, there were 12 up-regulated and 8 down-regulated proteins or genes with high differential expression levels mainly related to the following pathways: Glutaminergic synapse; GABAergic synapse; DNA replication; Synaptic vesicle cycle; Cell adhesion molecules; Circadian entrainment; Alanine, aspartate and glutamate metabolism; Long-term depression; Leukocyte transendothelial migration.

*In the Z7M vs K7M* comparison group, there were 7 up-regulated and 8 down-regulation proteins or genes with high differential expression levels mainly related to: Salivary secretion; Complement and coagulation cascades; Insulin signaling pathway; Tuberculosis; *Staphylococcus aureus* infection; Nitrogen metabolism; Systemic lupus erythematosus; Fatty acid biosynthesis; Steroid biosynthesis; Asthma.

*In the Z7M vs K2M* comparison group, there were 14 up-regulated and 6 down-regulation proteins or genes with high differential expression levels mainly related to: GABAergic synapses; Long-term depression; Glutaminergic synapses; Synaptic vesicle cycles; Retrograde endocannabinoid signaling; Circadian entrainment; Amyotrophic lateral sclerosis; ECM-receptor interaction; Morphine addiction; Protein degradation and absorption.

### 3.5 Overall proteomics results

**Fig. 18:**
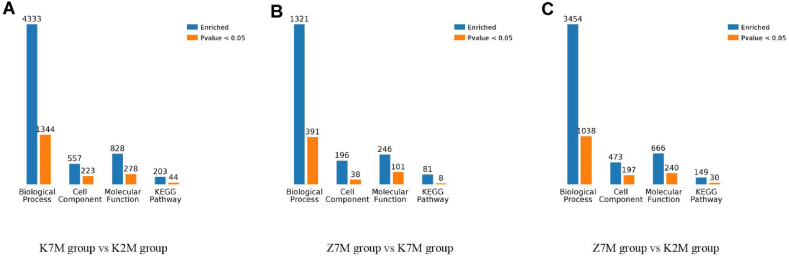
Total numbers of differentially expressed proteins in GO enrichment and KEGG pathways in the three comparison groups. (A) K7M vs K2M; (B) Z7M vs K7M; (C) Z7M vs K2M.

**Fig. 19:**
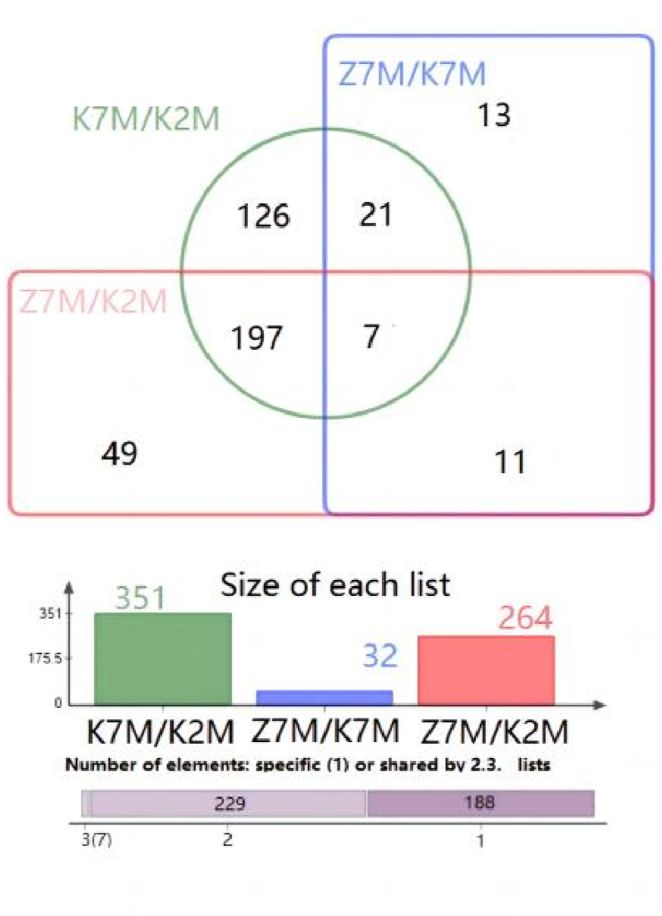
Summary of GO enrichment results. There were 351 differential proteins in the K7M vs K2M group, 264 differential proteins in the Z7M vs K2M group, and 52 differential proteins in the Z7M vs K7M group. It was found that only 7 proteins showed significant differences among all 3 experimental groups.

**Fig. 20:**
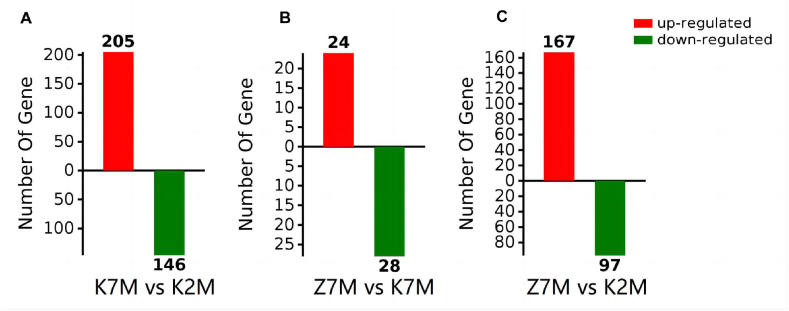
Number of up-regulated and down-regulated proteins in each comparison group. (A) K7M vs K2M; (B) Z7M vs K7M; (C) Z7M vs K2M.

The total number of differentially expressed proteins (1.5 fold increased or 0.66 fold decreased, p < 0.05) from GO analysis and KEGG pathway enrichment are shown in Fig. 18A −18C. In the K7M vs K2M comparison group there was a total of 4333 proteins relating to biological processes, and 1344 of these proteins showed a significant difference. There were 557 proteins relating to cellular components and 223 of these proteins showed a significant difference. There were 828 proteins relating to molecular functions and 278 of these proteins showed a significant difference. There were 203 proteins relating to KEGG pathways and 44 of these proteins showed a significant difference. In the Z7M vs K7M comparison group, there were 1321 proteins relating to biological processes and 391 of these proteins showed a significant difference. There were 196 proteins relating to cell components and 38 proteins of these proteins showed a significant difference. There were 246 proteins for relating to molecular functions and 101 of these proteins showed a significant difference. There were 81 proteins relating to KEGG pathways and 8 of these proteins showed a significant difference. In the Z7M vs K2M comparison group, there were 3454 proteins relating to biological processes and 1038 of these proteins showed a significant difference. There were 473 proteins relating to cell components and 197 of these proteins showed a significant difference. There were 666 proteins relating to molecular functions and 240 of these proteins showed a significant difference. There were 149 proteins relating to KEGG pathways and 30 of these proteins showed a significant difference. The results of the Venn diagram of the three comparison groups showed that the total numbers of proteins with a significant difference (p < 0.05, Fig. 19) were 351 in the K7M vs K2M comparison group, 52 in the Z7M vs K7M comparison group, and 264 in the Z7M vs K2M comparison group. The fact that the number of differentially expressed proteins (351) between K7M vs K2M was higher than between Z7M and K2M (264) suggests that the CHEA administration may have gone part of the way to restore the proteomics landscape to that of healthy young mice, but there was still a long way to go. However if this was the only effect, there should not be any differentially expressed proteins specific to the Z7M vs K2M group, they should be a subset of the K2M vs K7M differentially expressed proteins. However, there are 60 differentially expressed proteins in the Z7M vs K2M group which were not differentially expressed in K2M vs K7M, so something else is going on besides a restoration of the protein expression changes caused by aging. There were only 7 proteins with significant differences that were common to all three comparison groups. In the K7M vs K2M comparison group there were 205 up-regulated proteins and 146 down-regulated proteins (Fig. 20A). In the Z7M vs K7M comparison group, 24 proteins were up-regulated while 28 proteins were down-regulated (Fig. 20B). In the Z7M vs K2M comparison group, 167 proteins were up-regulated while 97 proteins were down-regulated (Fig. 20C).

## 4 Discussion

The technique of proteomics involves the study of protein expression on a large scale in an attempt to gain a deeper understanding of the mechanism of disease states or therapeutic interventions. In addition to comparing the expression levels of specific proteins between different groups, it can provide information on post-translational modifications, and protein-protein interactions. The concept was first proposed by Marc Wilkins in 1994 [18]. Proteomic studies can not only provide a basis for all of the processes in living organisms, but can also provide a deeper understanding of the causes and treatments for many diseases. By comparing the proteome between normal and pathological states, we can identify “disease-specific protein molecules", which could be molecular targets for new drugs, or provide molecular biomarkers for the early diagnosis of various diseases. Therefore, proteomics research can be used in addition to genomics research to provide a powerful tool for finding new drugs.

The mechanisms behind the development of AHL are quite complex, and many aspects of the etiology remain poorly understood. Recent studies have listed several potential contributing factors, including ageing of the immune system (immunosenescence), chronic inflammation and oxidative stress resulting in cellular damage and loss of hair cells by apoptosis. Other sites in the inner ear may also contribute, including the stria vascularis and synapses [19]. Indeed, our findings in this study support the involvement of age-related immune dysfunction and synaptic degeneration in the pathogenesis of age-related hearing loss. Further study on the relationship between age-related systemic immune dysfunction and neurodegeneration could open up new treatments for presbycusis, for which there is no effective therapy at present [20].

This proteomic study used the cochleae extracted from a mouse model of AHL to show that many proteins showed significant differences, and these involved complex pathways and mechanisms. The use of CHEA as a preventative treatment also affected many proteins and pathways, such as immunosenescence, chronic inflammation and activation of cochlear macrophages, decrease in cell function and immune response, disrupted energy metabolism, and damage caused by reactive oxygen species (ROS), all of which are expected to increase with advancing age [21]. However, the ability of CHEA to protect cell function and energy metabolism, enhance immunity, prevent ROS damage, and resist apoptosis could explain its effect on AHL. These findings further support previous studies on the related etiology and mechanisms of AHL [[22], [23], [24]] including our own studies [[5], [6], [7], [8],^10^,^11^].

In addition, this proteomic study also found other new effects of CHEA such as improving cardiovascular function, resisting bacterial infection or allergy, and preventing and treating senile diseases, which also fits with the hypothesised mechanisms of CHEA. The following are some examples of the biological effects and up-regulated proteins produced by CHEA treatment in this mouse model of AHL.

We found that CHEA could promote neuroprotein synthesis and energy metabolisms, as seen by the upregulation of NEFM (ID:P08553), which plays an important role in neuronal gene transcription and biomechanics and the long-term growth mechanism of axons [25].

Titin, encoded by the gene TTN, is involved in specific types of muscular dystrophy and cardiomyopathy [26,27]. Up to now titin has been mainly implicated in muscle function as it acts as a molecular spring that is responsible for the passive elasticity of muscles. Titin might play a role in AHL because it interacts with many other proteins and downstream signaling pathways [28], but further research is needed to identify exactly why this might be the case.

Another protein upregulated by CHEA is CKM (ID:A0A0J9YKD4), an active enzyme in mitochondrial energy metabolism [29]. Mitochondria are critical for hair cell function, which may explain why upregulation of CKM helps protect against ARHL [30].

CA1 is an isoenzyme of the carbonic anhydrase (CA) family, which regulates the pH of body fluids [31]. CA1 and CA2 have been found to be highly expressed in the striae vascularis and fibrocytes [32] and the spiral ligament [33] within the cochlea.

SA3K is a serine protease inhibitor (also known as Serpina3K), which plays an important role in inhibiting ROS production, protecting against oxidative damage, and anti-apoptosis. It has been shown to exert a neuroprotective function after traumatic brain injury [34], and its protective effect in a rat retinopathy model suggests antioxidant activity [35]. Indeed, it has been shown to protect retinal cells against oxidative stress-induced cell death [49]. It may play a similar protective role in the inner ear, thus its upregulation by CHEA could contribute to the observed protective effect.

The proteomics study found that CHEA could protect and enhance immune defense, which may protect against AHL and inhibit apoptosis of immune cells. This decrease in immune system activity is an important factor in age-related organ dysfunction. Senescence of the immune system also plays an important role in age-related degeneration of the inner ear. Examples of immune related proteins up-regulated by CHEA, included BPI fold-containing family B member 1 (Bpifb1, ID: Q61114), cathelicidin antimicrobial peptide (Camp, ID: P51437), lysozyme C-1 (Lyz1, ID:P17897), complement component 9（C9, ID:A0A0R4J032), ficolin-2 (Fcn2, ID:O70497), uroporphyrinogen-III synthase (Uros, ID: P51163), and deleted in malignant brain tumor 1 protein（Dmbt1, ID:A0A140LI59).

Bpifb1 is a member of the BPI-fold-containing family, which acts as an immune protective molecule [36]. Camp is a natural antibacterial peptide, which is widely distributed in the human body (especially in the mucus layer) where it plays an important role in the host innate defense against microbial pathogens [37]. Lyz1 is type of lysozyme, with high antibacterial activity due to its positive charge. Lysozyme plays an important role in innate immunity and provides protection against bacteria, viruses and fungi [38]. C9 is an important component of the complement system, which can combine with other complement components, C5b, C6, C7 and C8 to form a MAC (membrane attack complex), which binds to the surface of microorganisms to kill them [39]. Ficolin 2 (Fcn2) is a sugar-binding protein with collagen-like and fibrin-like domains. Ficolin plays a role in the lectin pathway to activate complement in innate immune responses. In addition to clearing pathogenic bacteria, fungi, and viruses, Fcn-2 also has roles in host homeostasis, including the clearance of apoptotic cells [40]. Uroporphyrinogen III synthase (Uros) is an important enzyme in the heme biosynthesis pathway [41] with roles in innate immunity [42]. Dmbt1 is an innate immune protein involved in mucosal protection, and regulates epithelial differentiation and inflammation. Dmbt1 can stimulate alveolar macrophage migration, suppress the neutrophil oxidative burst, and activate the complement cascade, thus suggesting an important role in the regulation of inflammatory responses [43]. One common feature of all these immune related proteins is that they have been implicated in innate immunity, which has not previously been strongly implicated in the etiology of AHL. This finding may provide clues for researchers investigating new therapeutic approaches for AHL.

The limitations of this study were as follows. There were no audiograms (ABRs) obtained from the actual mice from which the proteins were extracted. Even fully inbred wild type mice are not identical and random mutations affecting the inner ear could arise without being noticed. There was no analysis of CHEA treatment at an earlier age (which would have been a Z2M group). There was only a small number of mice when sex is taken into account, which means that differences in protein levels seen only in male or only in female mice were unlikely to be identified.

## 5 Conclusion

To sum up, we found that CHEA could regulate several proteins and genes related to various pathways, including synaptic molecular transmission, cellular energy metabolism, immune defenses, anti-oxidant defenses, anti-apoptosis, etc. The findings provide strong evidence to explain the mechanisms of CHEA, which had already been clinically proven to prevent and treat AHL and other types of sensorineural hearing loss. Among these mechanisms, stimulating an improvement in immune defense is a new highlight of this study.

It is known that AHL mechanisms are quite complex, including contributions from oxidative stress, ROS damage, gene variations and inheritance, microcirculatory disorders in the inner ear, immune dysfunction, arteriosclerosis, hypertension, diabetes, aging and degeneration of inner ear organs, environmental noise, ototoxic drugs, infection and inflammation [44]. This proteomics study found that there were many different proteins with statistically significant differences between each group, which involved multiple and complex regulatory pathways. It is impossible to explain them all clearly and in detail in only one publication, so they remain to be investigated one by one in the future. This article is a preliminary summary of the results, with a focus on new discoveries about which proteins were upregulated by CHEA. In addition to discussing various proteins related to AHL mechanisms such as promoting neural growth, eliminating ROS, anti-apoptosis, we also highlight newly discovered proteins affected by CHEA which regulate and enhance immune function, improve cardiovascular function, and reverse age-related disorders. We suggest that not only has CHEA been proven over the past decade to prevent and treat AHL and regulate AHL-related mechanisms, but it also could prevent and treat other diseases related to aging.

## Funding

This research was supported by 10.13039/501100001809National Natural Science Foundation of China grant No.81973913, No.81774374, No.81373700, No.81260552. MRH was supported by US NIH Grants R01AI050875 and R21AI121700.

## Declarations

All manuscripts must contain the following sections under the heading 'Declarations':

• The data of this study has been uploaded to iProx, https://proteomecentral.proteomexchange.org/cgi/GetDataset?ID=PXD041998.
• Ethics approval and consent to participate:All procedures were in accordance with the ethical requirements of experimental animals of Guangxi University of Traditional Chinese Medicine (Approval No.: IACUC 20161015).
• Consent for publication:Yes.
• Availability of data and materials: Yes.

## CRediT authorship contribution statement

**Weijun Xuan:** Writing – original draft, Methodology, Investigation, Funding acquisition, Formal analysis, Data curation, Conceptualization. **Liyi Huang:** Formal analysis. **Yi Xuan:** Formal analysis. **Sizhong Chen:** Formal analysis. **Junbo Tang:** Investigation. **Yulong Wei:** Resources, Methodology. **Xu Pan:** Investigation. **Michael R. Hamblin:** Writing – review & editing, Supervision.

## Declaration of competing interest

Weijun Xuan reports financial support was provided by 10.13039/501100001809National Natural Science Foundation of China. Michael R Hamblin reports financial support was provided by US NIH. Weijun Xuan has patent issued to Weijun Xuan. If there are other authors, they declare that they have no known competing financial interests or personal relationships that could have appeared to influence the work reported in this paper.

## Abbreviations

AHL: age-related hearing loss
TCM: traditional Chinese medicine
FDR: False discovery rate
FC: Fold change
CHEA: compound healthy ear agent
KEGG: Kyoto Encyclopedia of Genes and Genomes
GO: Gene ontology
SPF: Specific pathogen free
ROS: reactive oxygen species
GABA: Gamma-aminobutyric acid
Nefm: Neurofilament medium polypeptide
Ttn: Titin
Ckm: Creatine kinase M-type
CA1: Carbonic anhydrase 1
Serpina3k: Serine protease inhibitor A3K
Bpifb1: BPI fold-containing family B member
Camp: Cathelicidin antimicrobial peptide
Lyz1: Lysozyme C-1
C9: Complement component C9
Fcn2: Ficolin-2
Uros: Uroporphyrinogen-III synthase
Dmbt1: Deleted in malignant brain tumor 1 protein
